# Access to Tuberculosis Services for Individuals with Disability in Rural Malawi, a Qualitative Study

**DOI:** 10.1371/journal.pone.0122748

**Published:** 2015-04-01

**Authors:** Lisbet Grut, Lifah Sanudi, Stine Hellum Braathen, Thomas Jürgens, Arne H. Eide

**Affiliations:** 1 SINTEF Technology and Society, Oslo, Norway; 2 Reach Trust, Lilongwe, Malawi; 3 LHL's International Tuberculosis Foundation (LHL International), Oslo, Norway; Universita degli Studi di Roma Tor Vergata, ITALY

## Abstract

Tuberculosis occurs in all populations, but with higher prevalence in poor contexts. Vulnerable groups, including individuals with disability, run a particular risk due to poorer access to information and health services. Studying access to tuberculosis services for vulnerable groups in poor contexts may provide useful insight into the quality of such services in low-income contexts. This article aims to present a contextual understanding of access to tuberculosis services for people with disabilities in one district in southern Malawi. A qualitative method with semi-structured interviews and site observations was applied. In all, 89 participants were interviewed: 47 persons with disability, 11 parents/guardians of youths with disability, and the remaining 31 comprising eight health workers, four community rehabilitation assistants and volunteers, and 19 leaders in the community.Our main findings are that lack of information and knowledge, and considerable confusion related to tuberculosis, its cause and how to protect oneself, are major barrier to accessing services. Disease awareness and personal risk perception are key factors in this regard. Further findings concerns the pathways to tuberculosis related health services, in particular having a test and completing the treatment. The combination of lack of knowledge and barriers in accessing tests implies substantial availability and access problems.It is of importance to understand the combined impact of individual, social, contextual, and systems barriers to fully address the complexity of accessing tuberculosis services for vulnerable groups in poor populations. Lack of disability specific strategies in the local health services may be part of the reason why individuals with disability to not access such services.

## Access to Tuberculosis Services for Individuals with Disability in Rural Malawi

Tuberculosis (TB) occurs in all populations, but with higher prevalence among the poorest [1,2]. Equity in health requires that all individuals and groups have access to health services of good quality, and that services are provided according to individual needs [3,4,5]. In order to achieve equity in health, there is increasing empirical support for combining broad prevention and service programmes with specific measures directed towards vulnerable groups [6]. The way people with disabilities are served should be seen as a useful proxy for the quality of a health delivery system [6]. Thus, exploring the experiences of people with disabilities in accessing TB services is highly relevant for generating knowledge on the quality and availability of these services in low-income countries.

Poverty represents the most serious obstacle to accessing health services and to act according to instructions and information given by health personnel. Poverty reflects deficiency in many dimensions of a person's life, with each dimension potentially having an impact on all the others. Poor people often have reduced ability to describe symptoms of illness, staying healthy, and complying with the demanding lengthy regimen of TB treatment. Furthermore, cultural belief systems may colour the decisions on health matters as much as modern knowledge of health, disease and treatment [7,8].

Malawi is a poor country with a serious HIV epidemic which has had a large negative impact on TB control services in the country. TB case notifications have risen from 5000 per year in 1985 to more than 25 000 per year up to 2007 [9]. Good data on TB incidence and prevalence in Malawi is difficult to produce. A synthesis of literature on TB in Malawi estimates new case notifications to be around 50 000 new cases per year [10]. Comorbidity of HIV and TB has previously been estimated to 70% [9]. The Government of Malawi has since 1999 responded to this through the Malawi National Tuberculosis Control Programme, including TB testing of all HIV positive [11].

For HIV and TB the benefits for integration are well documented and published [12]. While Malawi is known for having an innovative and efficient HIV programme, studies on the integration of monitoring HIV and TB have however pointed to several implementation challenges [13,14]. In particular, there are access problems for the rural and poor population [10].

In this article we will explore access to TB services for people with disabilities who are living in a resource poor context; what do they know about symptoms and protection and what kind of barriers do they face when they access TB services?

## Method

Qualitative methods with semi-structured interviews and site observations were applied [15,16]. A qualitative design is suitable when studying complex issues, such as what something means to people or why they act in certain ways [17,18]. Participants were selected by purposive sampling. Efforts were made to ensure variation regarding adults of both genders, different age groups, types of impairments, and professional and occupational background. The entry point to identifying people with disabilities, traditional leaders and religious leaders was the Malawi Council for the Handicapped (MACOHA). Additional persons were identified by snow-ball sampling. The health workers were identified through the health clinics. Inclusion of new participants stopped at point of saturation. The field work was conducted in Machinga district in southern Malawi during three weeks in June 2011. The interviews were carried out by the first and second author of this article together with three field assistants. All interviews were carried out in the local languages. Hearing impaired participants were interviewed in sign language. In all, 89 participants were interviewed with approximately six of each informant group (Table 1): 47 people with disabilities older than 18 years and 11 parents/guardians of youth 14–18 years old with disabilities; eight health workers involved in the provision of health services in general, and HIV and TB services in particular, at primary and secondary level; four community rehabilitation assistants and community volunteers; eight religious leaders, 10 traditional leaders, and one former local ward councillor who also had a physical impairment.

**Table 1:** Participants in the study.

Participants in the study		
Type of participant	Male	Female
***Parents or guardians* (Condition of child/ward)**		
Physical disability		3
Epilepsy	1	1
Albino		2
Blind		2
Deaf and dumb		1
Learning difficulties		1
**Total**	**1**	**10**
***People with disabilities***		
Physical disability	13	12
Deaf and Dumb	2	4
Hearing impairment	0	3
Albinism	0	3
Visual impairment	2	0
Learning Difficulties	1	0
Blindness	5	2
**Total**	**23**	**24**
***Health workers (KI)***		
Medical Assistants	2	0
Health Surveillance Assistants	1	0
TB Officer	2	0
ART Coordinators	1	0
Nurse	1	0
HTC coordinator	1	0
Social Welfare Officer	0	1
MACOHA Staff	2	1
Religious leaders	8	0
Village headmen	10	0
Former councillor	1	0
**Total**	**29**	**2**

Development of the interview guide was inspired by Kleinman [17]. Participants with a disability were asked to tell about their everyday life, impairments and activity limitations, illness experiences, conditions (symptoms) that would compel for health care seeking, experiences of accessing health care, the type of care and support received at the facilities, and their experiences and satisfaction with the services received. Health care workers, social workers, religious- and village leaders were probed about provision of health care for people with disabilities, the skills and qualifications the health services have to serve their needs, and challenges of providing optimal health care related to TB and HIV to people with disabilities. Direct observations were done in and around the interview settings, mostly inside the homes of the participants and at health facilities. The interviews were recorded, transcribed verbatim, and translated into English. The transcripts were imported into MaxQDA software, and coded in accordance with the pre-developed themes and sub-themes of the interview guide. The transcripts were re-read in order to identify new categories and findings.

A thematic analysis was done looking for common as well as divergent experiences and opinions across the interview scripts. Ethical clearance was sought and obtained from the Stellenbosch University Ethics Board and the National Health Sciences Research Committee (NHSRC) of the Ministry of Health in Malawi.

## Results

### Knowledge about TB

Most of the participants with disabilities had picked up that a persistent cough may indicate TB. To the question on what they know about TB, a common answer was that if they are coughing for more than three weeks they should go for a TB test. Some of them explained that every now and then they felt sick and suffered from cough and chest pain because of the cough. But because the coughing stopped after a week or two, they used to buy pain killers at the local shop and did not seek medical services at the health clinic.

Some believed that TB is not contagious. Some believed that TB is a result of AIDS only, and because of an HIV negative status, they had never considered the possibility of being infected by TB. This is what one blind man replied to the question of being tested for TB:

They tell us that when someone is infected with HIV, TB comes out because TB is a disease for those with AIDS. In my case I never suffered from AIDS and they said that I never need to go for TB testing.

However, a few of the people with disabilities said they had knowledge on TB. A young woman with a physical disability had a family member who suffered from TB. The situation of her family member had shaped her conception about symptoms and precautions:

He lost weight and got thin, and coughed for a long time and was vomiting blood. We gave him a bottle to spit into and threw it away, and he was sleeping in his own bed. And other persons should not be very close to someone who is suffering from TB.

Another participant, a young man, had acquired similar information from many sources—health personnel, volunteers at MACOHA, village headmen and friends. He told that he had learnt that one should not share the bed with a person who is coughing, and when coughing one should not spit everywhere.

### Knowledge about TB tests

Most of the participants with disability (47 out of 58) said they had never taken a TB test, and they provided different explanations for this. Some did not perceive that they had ever needed a test as they had always felt healthy. Others told that they had gone to the hospital to be tested when they were ill, but the hospital had been out of medical equipment at the time, and they never tried again. Others again, told that they were not able to transport themselves to the hospital. The 11 participants with disability who said they had been tested for TB were tested because they had suffered from long lasting cough and fever. All of them were tested a few years back and none reported testing positive for TB.

Some of the participants were unclear about whether or not they had been tested for TB. It seems that they had not understood what kind of tests they had taken. This is illustrated in an interview with an HIV positive man (physical disability). He replied inconsistently to the question on TB testing. Early in the interview he stated that he had only been tested for HIV and never for TB. Later, the interviewer repeated the questions about TB testing. He then explained that he had been asked to submit sputum, and this test was negative:

I think it was last month when I felt very hot and after that I could feel cold again. They took a bottle and asked me to submit sputum and they asked me to stay for two days and on the third day they told me they had not diagnosed me TB positive.

When he was asked about whether the health personnel had explained to him the risk for TB because he was HIV positive, he replied:

They did not explain anything to me. I went to the hospital and I explained my problems to them. The officer wrote a note and after that I went to meet a medical officer. He took the form and read it and then he took drugs and gave me. I will not be honest with you if I told you that they explained to me. There was nothing else. They just told me to continue to take my medication.

A possible reason for the apparent confusion on TB tests may be that either the health personnel did not explain to him what kind of treatment he was given, or he did not understand the explanation given to him. In addition to the health services, MACOHA provides health related information to people with disabilities, but apparently not sufficient information on TB. An HIV positive visually impaired woman who used to attend local meetings arranged by MACOHA, said:

You may discuss an issue if you know about it, but when you don't know anything where will you start from?

As a HIV positive person she had received information about HIV, but never about the risk of TB connected to it.

### People are informed about HIV but not about TB

A total of 28 participants with disability had tested for HIV. Of these, five confirmed that they were HIV positive and receiving ARV treatment. One of them confirmed having taken a TB test. Two of them said they had never given sputum for a test, and two did not know whether they had been tested or not.

When asked about TB many of the participants seemed to mix the symptoms of TB and those of HIV, and thus confuse TB test with HIV test. While lacking information about TB, the participants seemed to be well informed about symptoms and precautions for HIV. Several of them revealed concern with HIV, including causes, signs and symptoms, prevention, as well as available treatment options; HIV appeared to be a common topic in daily conversations with friends.

This may reflect the emphasis placed on HIV information provision by different stakeholders at the expense of other illnesses, such as TB. A MACOHA volunteer explained that they have arranged many different activities during recent years targeting the welfare of people with disabilities. Health has been among the topics, including how to protect oneself from HIV, but they have not particularly focused on TB:

No, I can't say that we have a targeted programme of that nature; we are just looking at disability in general.

The participant's statement complies with the explanations from participants with disabilities. Both health and social workers and volunteers within the disability field said they have spent a lot of time informing about HIV/AIDS during recent years. On the ground in rural areas, there are more stakeholders taking HIV messages to the people than there are those providing information on TB and other diseases.

### Operational and logistical barriers

Among the factors that hamper access to TB diagnosis is lack of capacity at the nearest health facility. The service provider with the competence to examine and test for TB is not at the clinic on a daily basis. Some of the participants with disabilities said they refrained from seeking help at the clinic as they had previously been turned away because there were no personnel present or that the clinic could not attend to their particular problem on that specific day.

Several participants said that the clinics often lack diagnostic equipment for TB testing. The patients are then told to come back another day or they are referred to the district hospital. Accessing a health facility is strenuous for many people with disabilities, and requires more energy and resources than they can muster. As such, they often struggle or fail to comply with the referral. A health provider at a local health facility described the challenges of people with disabilities the following way:

The challenges are that we do not have sputum tests at the local health facility. When we suspect that he or she can have TB we give them sputum container to spit into, and leave it to their own to go the district hospital. It is a big challenge to a person with disability to be sent to the district hospital where the person has to wait for the sputum doctor and then have to come back to the district hospital to start medical treatment.

A participant who volunteers as a counsellor in a disability group confirmed that health services, particularly TB services, do not make deliberate attempts to reach people with disabilities in order to ease the access problems which they face. He said:

We have problems especially on health issues. I don't know if my friends have ever heard of people doing sputum collection and we don't have such activities here. Neither did we hear them saying 'we will be coming door to door to do sputum issues'.

While decentralization of services is a priority to increase access to health care, TB services seem not to be sufficiently decentralised in order to reach the marginalized and vulnerable groups in the communities.

### Reception by health personnel

It has been claimed that persons with disabilities experience stigmatisation by health personnel [19], but this was not found in the present study. The health personnel and social workers said that they treat persons with disabilities as anybody else and they give them priority when needed. According to the participants from MACOHA, there have been campaigns in Malawi to ensure and strengthen health personnel's attentiveness towards the needs of persons with disabilities. Interviews with persons with disabilities also confirm this. The participants with disabilities stated that they have been treated with respect by health and social workers, and they are often tended to before others at the health facility. Some admitted that problems sometimes arise when other patients become angry because they are prioritised in the queue.

### Disability not targeted

TB is not included in programmes that target people with disabilities and vice versa. Health service programmes targeting TB have not focused on people with disabilities and as such do not deliver health care that is tailor-made for their needs. The following quote from the interview with an ART officer illustrates this:

We have just integrated TB and HIV/AIDS in the past three or four, five months. Unfortunately, we were not looking at the physical disability when we were doing the screening.

This interview with the ART officer indicates that health services, in this particular area, have only recently taken the consequence of TB and HIV co-morbidity and the particular needs of people with disabilities seriously.

### Limitations of the study

The study has applied qualitative methods within a limited geographical area. It is therefore cautioned against interpreting the generated knowledge as representative for the population of disabled persons. We have further not collated the information from the participants with information from medical records and thus do not know for sure whether those who reported to be HIV positive have been tested for TB without knowing it.

Limited knowledge and access makes it difficult to find people with disabilities who have experiences with TB services. The findings are therefore partly related to access to health services in general, but with clear relevance also for specialised TB services. The study has provided insight into the experiences at individual and service level which is of importance for improving availability and accessibility of TB services for people with disabilities in a low income country, such as Malawi.

## Discussion

Barriers and facilitators to accessing TB services for persons with disabilities occur along the pathway to seeking health care, from contextual, social and individual factors influencing health seeking behaviour, to the encounter with health services, diagnosis and completion of treatment. Barriers identified through this study apply to the general population as well as to individuals with disability. We will however argued that the general access problems in a poor context have greater negative impact on vulnerable groups, such as individuals with disability, and that disability—specific barriers contribute to increase barriers to access health and TB services.

A main finding is that lack of information and knowledge, and considerable confusion related to TB, its cause and how to protect one self, is a major barrier. False and/or missing knowledge influence on people's health care seeking behaviour and is compromising the efforts to control TB. Hence, tackling this is important to protect the communities from TB.

How to protect oneself from TB in a resource poor setting is a difficult topic. In a hospital this is straight forward (i.e. basic infection control). But in the communities poor people are likely to live in poorly ventilated and overcrowded houses, and it may be potentially stigmatising to advice people to keep a distance to people who are coughing. Rather, one should strengthen sensitisation on knowledge on TB and positively encourage people who are coughing to get tested and treated.

Another main finding concerns the pathways to TB-related health services, in particular having a test and completing the treatment. Lack of knowledge is maybe the most critical contextual factor. Combined with a centralised structure of TB services, this implies substantial access problems for people with disabilities. Furthermore, the decentralised health services in the present study seem not to have equipment and sufficient competence to serve people with disabilities who need TB screening and treatment. Although Malawi has implemented pro-poor measures through the National TB programme and has made some progress in improving access, further measures are needed to improve case finding in poor areas.

This study indicates that information about HIV has penetrated also into rural and remote areas. In itself, this is positive, but it may be that health promotion activities and programmes in this and similar contexts have been overloaded with HIV information at the cost of other serious health problems, such as TB which is the main cause of death for HIV positive in Africa. It evidently adds to the confusion that TB and HIV are highly correlated, but it seems that information about TB has either been limited or simply ignored. This is an important message to health authorities responsible for health promotion in rural and resource poor areas. To what extent the concentration on HIV is due to the activities and interests of international organisations, is a relevant question to rise.

A further indication of quality problems is the finding that HIV positive participants report not to have been tested for TB, which is a requirement according to international as well as national recommendations [11]. This finding is also in contrast to the intentions behind the integrated HIV/TB services in Malawi [13]. Bearing in mind that this is a qualitative study within one rural area, one can of course not draw any conclusions further than indicating a possible problem with implementing TB tests for HIV positive persons. This concurs with the implementation problems reported by WHO and it is in line with Harris et al. [14] who claim little evidence for integration of TB-HIV intervention on the ground in sub-Saharan countries, Malawi included.

As the benefits for integration of TB-HIV services are well documented and published [12] such mode of delivery should require urgent attention so that individuals who have co-morbidity are diagnosed and treated at the same time and at the same facility.

Integration of TB services with other services could also be a solution, but this is more problematic as there is no simple point of care test. It is not feasible to run a microscope and maintain its quality in all health care settings. A possible option would be to train staff in TB and to collect sputum samples, and preparing smear slides, including a courier system to transport the slide sample to microscopy centre and deliver the results, so that the patients does not need have to travel. TB treatment demands a six month treatment which, technically, demands supervised daily intake of drugs. This will be even more challenging for patients with mobility challenges.

People with disabilities face a spectre of different barriers, both general and impairment specific, that affect their daily life activities in general and accessing health services in particular. They thus require more and often a variety of support from families, local communities and public health and social services. Lack of disability specific strategies in the local health services may be part of the reason why individuals with disability do not access TB services. The ability of TB services to target and reach vulnerable groups and individuals with disability in particular can be regarded as an indicator of quality of health services and a necessary strategy to reach equity in health in poor contexts.

## Supporting Information

S1 Data (PDF)
