# Correction: Access to Tuberculosis Services for Individuals with Disability in Rural Malawi, a Qualitative Study

**DOI:** 10.1371/journal.pone.0127607

**Published:** 2015-04-30

**Authors:** The PLOS ONE Staff

The affiliation for the second author is incorrect. Lifah Sanudi is affiliated with REACH Trust, Lilongwe, Malawi, not with SINTEF Technology and Society, Oslo, Norway.

The publisher apologizes for the error.
