# Supplementary material for: Access to Tuberculosis Services for Individuals with Disability in Rural Malawi, a Qualitative Study
Source: PLoS One. 2015 Apr 1;10(4):e0122748. doi: 10.1371/journal.pone.0122748 (PMC4382312; doi:10.1371/journal.pone.0122748)
Supplement: S1 Data — (PDF) [file pone.0122748.s001.pdf]

Excerpts from the transcripts that are not included in the Result section of the article are *italicised* and written *in colour.*

Results

Knowledge about TB

Most of the participants with disabilities had picked up that a persistent cough may indicate TB. To the question on what they know about TB, a common answer was that if they are coughing for more than three weeks they should go for a TB test.

(Excerpt from an interview, citation not included in the article)
Int.: do you know anything about TB?
Resp.: I have heard that when people start coughing up to the extent that they are spitting blood. It is a very stinging cough.
Int.: then, can you tell me how one gets contaminated from TB?
Resp.: I have heard that you can get it from smoking tobacco.
Int.: but how does TB spread?
Resp.: I don't know.
Int.: what about preventing it?
Resp.: do not smoke tobacco and do not eat food which the doctor tells you not to eat.
Int.: have you ever decided to go for a TB test?
Resp.: no.
Int.: and why have you never decided this?
Resp.: because I have never suffered from it and I have never coughed that severe.

This is what one blind man replied to the question of being tested for TB:

Int.: What do you hear about TB?
Resp: I just hear from other people that one coughs persistently.
They tell us that when someone is infected with HIV, TB comes out because TB is a disease for those with AIDS. In my case I never suffered from AIDS and they said that I never need to go for TB testing.
Int: What do they say TB is?
Resp: I don't know the type of germ that is.
Int: Have you received any messages concerning TB either from people or other sources?
Resp: Maybe from the radio. I just hear of such messages but I don't utilize such messages because I have never suffered from that.
Int: What messages do they give out?
Resp: They say there is AIDS but is TB not as a result of AIDS? They say it is a disease for those with AIDS but I don't know much about it madam and in my case I have never suffered from that.

Int: It is not that we are asking whether you had suffered form that but we just wanted to know whether you have access to such information.
Resp: I just hear the messages from the radios but I do not take the messages seriously.
Int: Why don't you take the messages seriously?
Resp: It is because I have not experienced that.
Int: Apart from radios or from your friends, is there somewhere else you heard about these issues?
Resp: I don't know other sources because I just stay here at home, I don't go anywhere else.
Int: What about HIV issues, what would you say about that?
Resp: I just hear that from people but I cannot manage to explain it.
Int: Have you ever decided to have an HIV test?
Resp: I have never thought of that.
Int: Then, what do you know about HIV and AIDS and TB?
Resp: I have been hearing that there is HIV that kills a lot of people in our community.
Int.: how does one get HIV?
Resp.: it is through sex and razor blades.
Int.: From where do you get this information?
Resp.: It is through the radio and Macoha.

A young woman with a physical disability had a family member who suffered from TB. The situation of her family member had shaped her conception about symptoms and precautions:

Int.: Have you ever heard about TB?
Resp.: yes I know something about TB because my (family member) suffered from it for a year.
He lost weight and got thin, and coughed for a long time and was vomiting blood.
And I remember him having stayed at the health facility for close to two months before they were released to finish the treatment at home.
Int.: what did you to then?
We gave him a bottle to spit into and threw it away, and he was sleeping in his own bed. And other persons should not be very close to someone who is suffering from TB.
Int.: have you ever decided to take a TB test?
Resp: yes when my (family member) was diagnosed with TB we were asked by the health workers to go for a test. I went to submit sputum and I tested negative.
Int.: who made the decision to get tested?
Resp.: at first it was the doctor who asked my (family member) to tell me that I needed to get tested and then after learning from my fellows that they all were tested I asked the health worker that I wanted to get tested.
Int.: how were you treated at the health facility then?

Resp.: It was good.

Another participant, a young man, had acquired similar information from many sources – health personnel, volunteers at MACOHA, village headmen and friends. He told that he had learnt that one should not share the bed with a person who is coughing, and when coughing one should not spit everywhere.

(Excerpt from the interview, citation not included in the article)
Int.: do you know how to protect yourself from contaminating TB?
Resp.: yes I do know because my (family member) suffered from it.
Int.: what did you do?
Resp.: we took him to the hospital and he got medicine. He suffered for one year and then he died.
Int.: did you think he could transfer the TB to you?
Resp.: yes we did.
Int.: how did you get this information?
Resp.: I get it from Macoha and meetings with the group village headmen and the day they came to open the boreholes they also spoke about AIDS.
Int.: have you ever decided to go for a TB test?
Resp.: I have never decided that. I went for a HIV test and was found negative.

Knowledge about TB tests
Most of the participants with disability (47 out of 58) said they had never taken a TB test, and they provided different explanations for this. Some did not perceive that they had ever needed a test as they had always felt healthy. Others told that they had gone to the hospital to be tested when they were ill, but the hospital had been out of medical equipment at the time, and they never tried again:

(Excerpt from an interview (woman with hearing impairment), citation not included in the article)
Int: Who accompanied you to the hospital?
Resp: I went with my (family members)
Int: When you reached to the hospital did they help you?
Resp: I was welcomed with comfort
Int: How far did they assist you?
Resp: A doctor did not want to give me any treatment because they had no medical treatment on that day.
Int.: did you go again?
Resp.: We never went again because it is too far away and we do not have the means for transport.

(Excerpt, woman physical disability, citation not included in the article)
Int.: Have you ever had a persisting cough?

Resp: I have been feeling pain in my chest and ribs.
Int.: Have you ever decided to go for TB test?
Resp.: I went to the health facility for TB test and they told me that their equipment is very expensive. None can ask for a TB test, but if the health worker feels that you need one they can do a test.
Int.: Did you ever go again?
Resp.: I never have gone again for a test.

Others again, told that they were not able to transport themselves to the hospital.

(Excerpt from an interview with the guardian of a young girl with a physical disability, citation not included in the article)
Int.: have you ever decided to take her for a TB test?
Resp.: No, because we do not have the means for transport. She is very heavy as she is fat and it is not easy for us to carry her even in a wheelchair.
Int.: have you ever decided to take her to a HIV test?
Resp.: no, like me she has never gone for that test, but maybe we will go when the mobile clinic comes here.

The 11 participants with disability who said they had been tested for TB were tested because they had suffered from long lasting cough and fever. All of them were tested a few years back and none reported testing positive for TB.

Some of the participants were unclear about whether or not they had been tested for TB. It seems that they had not understood what kind of tests they had taken. This is illustrated in an interview with an HIV positive man (physical disability). He replied inconsistently to the question on TB testing. Early in the interview he stated that he had only been tested for HIV and never for TB. Later, the interviewer repeated the questions about TB testing. He then explained that he had been asked to submit sputum, and this test was negative:

Int.: have you ever been tested for TB?
Resp.: No I have never been tested.
Int.: do you know anything concerning TB, like what it is about or any other message about TB?
Resp.: No, I have never heard about that.
Int.: not even from the hospital?
Resp.: to be honest I cannot say I have much interest to hear everything they say at the hospital.
Int.: why do you not be interested?
Resp.: it is because of my mobility problems that are preventing me to get access to all that information. But it would do us some good if I heard about that.

> *Int.: have you ever suffered from cough that lasted for a while?*
> *Resp.: yes.*
> *Int.: can you tell me about that?*
> *Resp.:* I think it was last month when I felt very hot and after that I could feel cold again.
> *Int: what did you do to it?*
> *Resp.: I went to the hospital and* they took a bottle and asked me to submit sputum and they asked me to stay for two days and on the third day they told me they had not diagnosed me TB positive.

When he was asked about whether the health personnel had explained to him the risk for TB because he was HIV positive, he replied:

> They did not explain anything to me. I went to the hospital and I explained my problems to them. The officer wrote a note and after that I went to meet a medical officer. He took the form and read it and then he took drugs and gave me. I will not be honest with you if I told you that they explained to me. There was nothing else. They just told me to continue to take my medication.

A possible reason for the apparent confusion on TB tests may be that either the health personnel did not explain to him what kind of treatment he was given, or he did not understand the explanation given to him. In addition to the health services, MACOHA provides health related information to people with disabilities, but apparently not sufficient information on TB.

An HIV positive visually impaired woman who used to attend local meetings arranged by MACOHA, said:

> *Int.: so, what is the name of your group?*
> *Resp: it is (name omitted from the citation). This is our fourth year we have this group. Whenever we go to certain organisation to ask for support they refer us back to Macoha because they say we are under them.*
> *Int.: during some of your meetings, does it happen that you discuss issues of TB?*
> *Resp.: no we don't.*
> You may discuss an issue if you know about it, but when you don't know anything where will you start from? *Would you just start to discuss such an issue? Unless if you were trained. Then, when you are back you can invite others to teach them what it is all about.*

As a HIV positive person she had received information about HIV, but never about the risk of TB connected to it.

> *(Excerpt not included in the article)*
> *Int.: what about your husband? What happened to him that made him go for the (HIV) test?*

Resp.: at first he refused, but finally he accepted when he became seriously ill with diarrhoea. And I told him "look the way you are feeling. What am I going to do" and he finally accepted to go.
Int.: how long did it take him to go for the test?
Resp.: it took about a year and he started receiving the drugs and is still on treatment.
Int: were you aware of HIV when you were going for the test?
Resp: yes they were teaching us that when we went to the training at the rehabilitation facility.
Int.: what did they say to you?
Resp.: they were teaching us that having HIV test is good and being diagnosed with HIV is not the end of life, but it helps you to remove your worries and when you have started taking ARVs all your worries are washed away and you have a healthy life and a prolonged life. So that encouraged me to go for the testing.
Int.: why did you not decide to go for the test before?
Resp.: before we were going to get married I told him that we should go for the test, but he wanted to wait. I told him that other people who were falling in love went for the test, but he refused. And since I wanted so to get married I just left it like that.
Int.: and would you explain to us what you experienced at the hospital?
Resp.: when I got to the hospital they did not give me a chance to speak. They just took me for the testing and thereafter they said to me "will you be strong if we tell you the results of the test?" and I told them that they should explain it to me. And they said I was diagnosed HIV positive.
Int.: have you ever been going for TB test?
Resp: no I have never been tested for that.
Int.: Do you know anything concerning TB?
Resp.: No I do not know anything about that.
Int: have you ever experiences cough and night sweat?
Resp.: when that happens that I am coughing and feel pain in my chest I have my children to buy medicine from the groceries.

People are informed about HIV but not about TB
A total of 28 participants with disability had tested for HIV. Of these, five confirmed that they were HIV positive and receiving ARV treatment. One of them confirmed having taken a TB test. Two of them said they had never given sputum for a test, and two did not know whether they had been tested or not.

When asked about TB many of the participants seemed to mix the symptoms of TB and those of HIV, and thus confuse TB test with HIV test. While lacking information about TB, the participants seemed to be well informed about symptoms and precautions for HIV. Several of them revealed concern with HIV, including causes, signs and symptoms, prevention, as

well as available treatment options; HIV appeared to be a common topic in daily conversations with friends.

This may reflect the emphasis placed on HIV information provision by different stakeholders at the expense of other illnesses, such as TB. A MACOHA volunteer explained that they have arranged many different activities during recent years targeting the welfare of people with disabilities. Health has been among the topics, including how to protect oneself from HIV, but they have not particularly focused on TB:

> *Int.: When it comes to TB issues, does Macoha have officers that are trained as TB counsellors?*
> Resp.: No, I can't say that we have a targeted programme of that nature; we are just looking at disability in general.
> *TB is only dealt with in governmental institutions. What Macoha does is to organise the meetings and chat with them. If they have any problems we refer them to the hospital.*
> *Int.: so it is only HIV, loans and assistive devices that are focused?*
> *Resp.: yes.*

The participant's statement complies with the explanations from participants with disabilities. Both health and social workers and volunteers within the disability field said they have spent a lot of time informing about HIV/AIDS during recent years. On the ground in rural areas, there are more stakeholders taking HIV messages to the people than there are those providing information on TB and other diseases.

Operational and logistical barriers to accessing TB test and treatment

Among the factors that hamper access to TB diagnosis is lack of capacity at the nearest health facility. The service provider with the competence to examine and test for TB is not at the clinic on a daily basis. Some of the participants with disabilities said they refrained from seeking help at the clinic as they had previously been turned away because there were no personnel present or that the clinic could not attend to their particular problem on that specific day.

Several participants said that the clinics often lack diagnostic equipment for TB testing. The patients are then told to come back another day or they are referred to the district hospital. Accessing a health facility is strenuous for many people with disabilities, and requires more energy and resources than they can muster. As such, they often struggle or fail to comply with the referral. A health provider at a local health facility described the challenges of people with disabilities the following way:

Int: there is a claim that access to TB and HIV tests for people with disabilities are difficult. Do you have any information about what is the problem and how one could solve this problem?
Resp.: The challenges are that we do not have sputum tests at the local health facility. When we suspect that he or she can have TB we give them sputum container to spit into, and leave it to their own to go the district hospital.
The problem is that it is a long distance to come here for some people to get tested. People make some delays. They feel it is too long distance for them to take specimen to the hospital. We can give them the sputum containers and ask them to go to the hospital. And some do go and even some that are on TB treatment go there. But then some say they will go tomorrow or maybe next week. So it is always difficult.
It is a big challenge to a person with disability to be sent to the district hospital where the person has to wait for the sputum doctor and then have to come back to the district hospital to start medical treatment.
It would have been better if they had established a TB microscope centre where people can have their TB results from here.
Int.: in that case, would you like it to be a mobile centre or should it be like a village centre or how should it be organised?
Resp.: If that could be a health centre with a TB microscope it could be good. But not a village centre because when we are visiting the villages we go there with a lot of activities. We cannot only go with one activity.

A participant who volunteers as a counsellor in a disability group confirmed that health services, particularly TB services, do not make deliberate attempts to reach people with disabilities in order to ease the access problems which they face. He said:

We have problems especially on health issues. I don't know if my friends have ever heard of people doing sputum collection and we don't have such activities here. Neither did we hear them saying 'we will be coming door to door to do sputum issues'.
Int.: have Macoha been involved in TB and HIV issues targeting people with disabilities in any way?
Resp: For TB I know nothing but for HIV we always advise people with disabilities to go for HIV testing so that they should know their status and they always go for the HIV test.
Int.: In that what you are saying would you say that Macoha never trained you about TB, only on HIV issues?
Resp: yes. There is no one that is trained on TB issues here. Partly we were trained in HIV and in AIDS it was not a full training.
Int: what happens when people have the HIV test and it turns out to be positive?
Resp.: they come themselves and tell us and we always advise them to go back to the hospital to start the treatment early. So they come to us and we counsel them to go back to the hospital.

Int.: have you any community programmes on HIV?
Resp: as for now, not any. But at times we have awareness campaigns on HIV issues.
Int.: are there any other organisations on TB and HIV?
Resp: there are no specific organisations on that.

While decentralization of services is a priority to increase access to health care, TB services seem not to be sufficiently decentralised in order to reach the marginalized and vulnerable groups in the communities.

Reception by health personnel

It has been claimed that people with disabilities experience stigmatisation by health personnel [29], but this was not found in the present study. The health personnel and social workers said that they treat people with disabilities as anybody else and they give them priority when needed. According to the participants from MACOHA, there have been campaigns in Malawi to ensure and strengthen health personnel's attentiveness towards the needs of people with disabilities. Interviews with participants with disabilities confirm this. The participants with disabilities stated that they have been treated with respect by health and social workers, and they are often tended to before others at the health Intity. Some admitted that problems sometimes arise when other patients become angry because they are prioritised in the queue.

Disability not targeted

TB is not included in programmes that target people with disabilities and vice versa. Health service programmes targeting TB have not focused on people with disabilities and as such do not deliver health care that is tailor-made for their needs. The following quote from the interview with an ART officer illustrates this:

Int.: first, would you like to introduce yourself and the programme?
Resp: I am a general clinician and also an Art coordinator and a HIV focal officer for DHO. Here we have running the ART programme since 2005 and by 2011 we have rolled out to all health centres that are providing maternity care. So since that time I have been here full time and most of the time on ART.
Int.: would you estimate during the past six months how often you have served people with disabilities?
Resp: We have just integrated TB and HIV/AIDS in the past three or four, five months. *With ART even TB is now done here. They can give sputum here if they cannot go to the TB office.*
Unfortunately, we were not looking at the physical disability when we were doing the screening.

When we examine a disabled person they still refer him to either the lab or the TB office. I will not be able to give you the exact data (on how many people with disabilities) because normally we are not looking at if they are disabled or not. But I can say that here are very few patients with disabilities. We have some with blindness and others who have deformities maybe having a physical disability and some have hearing problems. We treat them just as they come and do not have special clinics for such patients. If we could just estimate it could be a very low figure, maybe less than 5% who have a disability.
Int.: when they come, what sort of problem do they represent?
Resp.: it varies from individual to individual. But most present transport problems. We see a lot of people with disabilities that are missing their appointments and most of the people with disabilities say that they had no means of transport.
Int.: on TB and HIV you said it is a number of patients who are coming to receive the treatment. How would you estimate how people with disabilities access TB or HIV treatment?
Resp.: we have just integrated from this year. Previously they used to go to the TB department. We may have more than one but we were not so seriously trying to monitor the disabled ones. But I know one case with a blind man whom I found on TB treatment.

This interview with the ART officer indicates that health services, in this particular area, have only recently taken the consequence of TB and HIV co-morbidity and the particular needs of people with disabilities seriously.
